# Culm cell-wall compositions of tribes Bambuseae and Olyreae from the Brazilian Atlantic Forest: Quantitative data from monosaccharide and oligosaccharide profiling and pectin/hemicellulose ratio

**DOI:** 10.1016/j.dib.2020.106078

**Published:** 2020-07-25

**Authors:** Marco A. Tiné, Michele Silva, Maria T. Grombone-Guaratini

**Affiliations:** a Núcleo de Pesquisa em Fisiologia e Bioquímica, Instituto de Botânica, São Paulo, Brasil, C.P. 68041, 04301-902, São Paulo SP, Brazil; b Núcleo de Pesquisa em Ecologia, Instituto de Botânica, São Paulo, Brasil, C.P. 68041, 04301-902, São Paulo SP, Brazil

**Keywords:** *Apoclada*, *Chusquea*, *Filgueirasia*, *Guadua*, *Parodiolyra*, hemicellulose, arabinoxylan

## Abstract

Bamboos are known for their economical, ecological, and cultural importance. The plants can be annual or perennial and can be herbs, shrubs or trees and can also show different growth habits. The cell wall is the main component of the mechanical properties of the tissues. Data set presented here contains the results of cell walls fractioning of culms from six neotropical bamboo species: *Apoclada simplex, Chusquea capituliflora, Filgueirasia arenicola, Filgueirasia cannavieira, Guadua tagoara, Merostachys riedeliana and Parodiolyra micrantha*. The cell walls were fractionated with oxalate and increasing NaOH concentrations sequentially. The yield and the monosaccharide compositions showed a small amount of pectin as expected for Poaceae and arabinoxylan as the main hemicellulose. The digestion of the hemicellulose fraction with xylanase produced an oligosaccharide profile that could be used to compare the similarity of the arabinoxylan from different species without identifying each individual oligosaccharide. Our data showed that the differences in cell wall composition do not vary according to the growth habit, but are in close association with the phylogenetic relations within the family. The differences in load capacity in plants with different habits (trees and herbs, for example) are more associated with the amount of support tissues than with different cell wall compositions. The importance of evaluate the cell wall of tropical bamboo species aimed at improving resources for biotechnology was discussed by Tine et al. 2020 [1].

## Specifications Table

**Table:** 

**Subject**	Environment Science
**Specific subject area**	Biochemistry
**Type of data**	Figure and Tables
**How data was acquired**	Cell wall fractionation from bamboo stems, acid and enzymatic hydrolysis, high performance anion exchange chromatography with pulse amperometric detection.
**Data format**	Raw and Analyzed
**Parameters for data collection**	Data was collected by Chromeleon software (Thermofischer). The chromatograms were integrated with the default parameters and the retention time was used to identify the peaks. Area was used to compare que amounts of monosaccharides and oligosaccharides.
**Description of data collection**	Culms segment of different bamboo species were dried, milled and extracted with ethanol. The ethanol insoluble residue was extracted with a sequence of increasing stringency (Oxalate, 0.1M NaOH, 1M NaOH and 4M NaOH. The monosaccharide composition of the extracted polysaccharides was obtained after hydrolysis with trifluoroacetic acid and analysis by anion exchange Chromatography with Amperometric pulse detection in a Carbopak PA-1 column with an isocratic eluent (16 mM NaOH). Fingerprinting of the arabinoxylan (4M NaOH fraction) was obtained by digestion of the 4M NaOH fraction with xylanase and analysis of the oligosaccharides in the same chromatographic system with an 88 mM isocratic elution.
**Data source location**	Site (18°6’23’’S;52°55’40’’W) Site (13°47’51”S; 47°27’30” W) Site (23°39’25”S; 46°37’41”W) Site (23°23′28"S; 46°25′24"W)
**Data accessibility**	Repository name: Zenodo.org Data identification number: DOI: 105281/zenodo.3820529
**Related research article**	Marco Aurelio Tiné, Michele Silva and Maria Tereza Grombone-Guaratini. Culm cell-wall compositions of tribes Bambuseae and Olyreae (subfamily Bambusoideae; family Poaceae) from the Brazilian Atlantic Forest. 10.1016/j.flora.2020.151596

## Value of the data

• The quantitative information of the yield of the soluble fractions of bamboo cell wall is key to understanding its potential use in biofuels. The qualitative analysis of these fractions and identification of the polysaccharides is described in [1], therefore the data presented here is complementary to the one presented in [1] and will allow raw estimations of the amount of biomass extracted from the bamboo walls.
• The data on the oligosaccharide's profiles will allow researchers to compare the structure of the arabinoxylan of these species with any other Poaceae. With the data reported in Table 2, regarding composition and retention times, a profile can be aligned to digestions in any laboratory that uses ion chromatography to compare the structure of arabinoxylan from different sources.
• This data will benefit researchers interested in bamboo and other grasses cell wall structure and phylogeny as well as those interested in feedstock screening for biotechnology.

## Data description

**Fig. 1:**
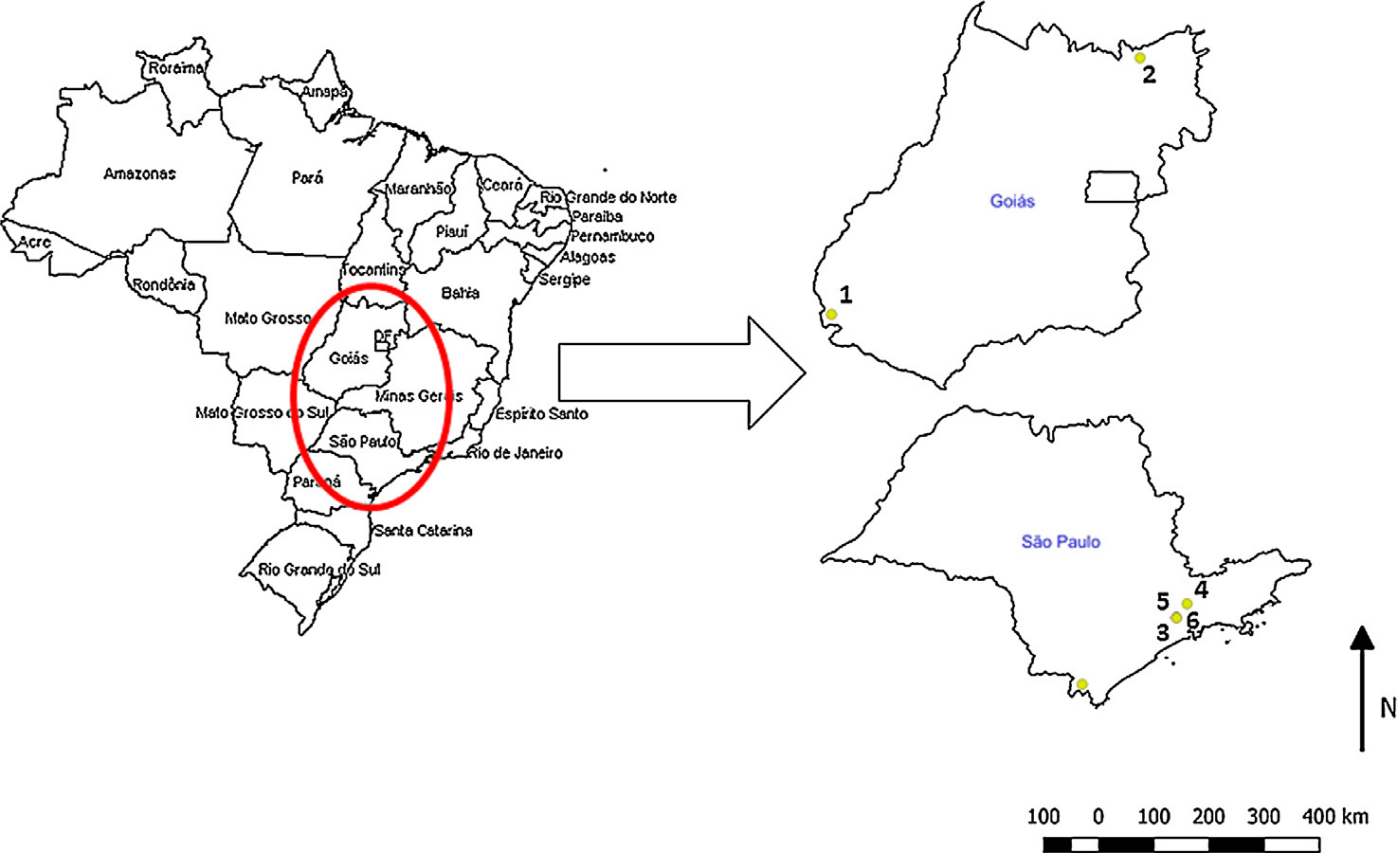
Location of the plots where bamboo were sampled, in the state of Goiás and São Paulo, Brazil. 1. *Filgueirasia arenicola*, 2. *F. cannavieira* 3. *Chusquea capituliflora* 4. *Guadua tagoara* 5. *Merostachys riedeliana* and 6. *Parodiolyra micrantha*. Map were prepared using ArcGIS

The dataset presented in this article show the location of The map of Brazil indicating the location of field sites in the state of Goiás where *Filgueirasia arenicola* (McClure) G.F. Guala and *F. cannavieira* (McClure) G.F. Guala were collected and in the state of São Paulo where *Chusquea capituliflora* McClure & L.B, *Guadua tagoara* (Ness) Kunth, *Merostachys riedeliana* Rupr. ex Döll and *Parodiolyra micrantha* (Kunth) Davidse & Zuloaga were collected (Fig. 1).

Table 1 estimates the amount of pectin and hemicellulose in the cell walls. The pectic fraction of the bamboo walls contains less than 10% of the wall as expected for poaceae, with hemicellulose constituting around half of the wall. Cellulose compromises the remaing of the insoluble residue. The exceptions are *Parodiolyra micrantha* with 10.7% of pectin and *Guadua tagoara* with only 35.4% of hemicellulose.

**Table 1:** Comparison of the yields of pectin and hemicellulose fractions of the bamboo walls. Averages of three biological replicas for each species.

Species	Pectin*	Hemicellulose⁎⁎	Ratio
*Chusquea capituliflora*	7.4	54.1	7.3
*Filgueirasia arenicola*	7.9	52.7	6.7
*Filgueirasia cannavieira*	6.7	54.3	8.2
*Guadua tagoara*	6.8	35.4	5.2
*Merostachys riedeliana*	8.0	52.7	6.6
*Parodiolyra micrantha*	10.9	46.4	4.3

⁎ =sum of the yield of the oxalate and the 0.1M NaOH fractions.

⁎⁎ = sum of the yield of the 1M and 4M fractions

Table 2 show the average percentage of the area of oligosaccharides produced by the action of xylanase on the hemicellulose of the species. The data shown is the average of nine digestions of three biological samples. The structure of the arabinoxylan is similar in all bamboo species and a multivariate similarity tree was used to compare the data [1], indicating the potential use of the technique for large scale screening of arabinoxylan in a wide rarieties of feedstocks even without the need of prior knowledge of the oligosaccharides structure. The great number of lines with “0” (which means oligosaccharides found only in sugarcane, used here as reference) point to the potential of the technique to compare the fine structure of the polymers across a wider range of taxonomic clades.

**Table 2:** Proportion of arabinoxylan oligosaccharides in the structure of the arabinoxylan present in the 4M fractions of the bamboo walls. Average of 9 digestions with xylanase.

**Peak n.**	**Ret. Time (min)**	***C. capituliflora***	***F. arenicola***	***F. cannavieira***	***G. tagoara***	***M. riedeliana***	***P. micrantha***
1	1.87	0.000	0.000	0.000	0.067	0.000	0.000
2	4.22	0.000	0.000	0.000	0.000	0.000	0.000
3	4.88	0.032	0.000	0.000	0.000	0.000	0.000
4	5.35	0.000	0.000	0.000	0.563	0.000	0.000
5	6.49	13.493	12.354	13.949	19.088	14.338	17.036
6	6.74	0.000	0.000	0.000	0.000	0.067	0.000
7	7.34	0.039	0.000	0.000	0.051	0.000	0.000
8	10.86	39.595	53.575	52.796	42.907	37.566	32.491
9	11.23	17.821	15.300	14.729	18.201	15.783	15.839
10	12.74	1.980	1.863	0.896	0.981	1.144	2.747
11	13.20	0.032	0.000	0.000	0.000	0.043	0.000
12	13.84	0.000	0.000	0.000	0.033	0.000	0.000
13	15.56	0.000	0.000	0.000	0.000	0.064	0.000
14	16.48	0.815	0.759	0.850	0.550	0.000	0.000
15	17.68	0.391	0.748	0.793	0.682	1.692	4.088
16	18.50	0.758	0.000	0.000	0.000	0.000	0.000
17	18.99	10.334	9.254	9.308	7.773	1.689	2.957
18	19.43	3.771	4.426	4.660	2.774	0.000	0.000
19	20.26	0.347	0.000	0.365	0.000	13.564	15.642
20	21.73	0.554	0.000	0.000	0.069	5.323	7.255
21	22.83	4.545	1.722	1.506	2.174	0.881	0.378
22	23.58	1.374	0.000	0.062	1.398	3.370	0.463
23	24.56	0.033	0.000	0.000	0.131	0.799	1.105
24	27.35	0.000	0.000	0.000	0.687	0.000	0.000
25	30.30	0.336	0.000	0.000	0.753	0.000	0.000
26	30.97	0.000	0.000	0.000	0.000	0.000	0.000
27	33.45	1.694	0.000	0.000	0.996	0.193	0.000
28	35.19	0.000	0.000	0.000	0.000	0.000	0.000
29	36.80	0.493	0.000	0.000	0.038	3.488	0.000
30	41.96	1.564	0.000	0.088	0.084	0.000	0.000

Arabinoxylan from sugarcane was used as standard. Oligos with “0” content was present only in sugarcane arabinoxylan.

## Experimental design, materials, and methods

The original raw material (middle third originating from an adult culm) was collected from three different populations clumps (biological replicate). The material was dried, milled and extracted with ethanol to remove low molecular weight metabolites. The alcohol insoluble material was extracted with oxalate and increasing concentrations of NaOH (0.1M, 1M and 4M) and the extracted material was dialyzed and freeze-dried. The monosaccharide compositions of the fractions were obtained by high performance anion exchange chromatography with amperometric pulse detector (HPAEC / PAD) (ICS-3000, Thermo), Carbo-Pac PA 1 column, with isocratic elution (16 mM, 0.9 mL.min^−1^ for 40 min) after hydrolysis with 2N trifluoroacetic acid. The elution profiles of monosaccharides were compared with those obtained for commercial monosaccharide standards. The main hemicellulose was arabinoxylan and the 4M fraction of the wall (richer in hemicellulose) was digested with xylanase. Oligosaccharide profiles were analyzed by high performance anion exchange chromatography with amperometric pulse detector (HPAEC / PAD) (ICS-3000, Thermo), Carbo-Pac PA 1 column, with isocratic elution (88mM at 0.9 mL.min^−1^ for 50 min) [2].

## Declaration of Competing Interest

The authors declare that they have no known competing financial interests or personal relationships that could have appeared to influence the work reported in this paper.

## Appendix Supplementary materials

Image, application 1
