# Supplementary material for: Culm cell-wall compositions of tribes Bambuseae and Olyreae from the Brazilian Atlantic Forest: Quantitative data from monosaccharide and oligosaccharide profiling and pectin/hemicellulose ratio
Source: Data Brief. 2020 Jul 25;32:106078. doi: 10.1016/j.dib.2020.106078 (PMC7412758; doi:10.1016/j.dib.2020.106078)
Supplement: Supplementary file 1 [file mmc1.docx]

Supplementary materials Supplementary material associated with this article can be found, in the online version, https://zenodo.org/record/3820529
